# Are biofuels sustainable? The EU perspective

**DOI:** 10.1186/1754-6834-1-9

**Published:** 2008-05-01

**Authors:** Sam Cockerill, Chris Martin

**Affiliations:** 1 Sam Cockerill Ltd, Link Hall, Wheldrake Lane, Crockey Hill, York YO19 4SQ, UK; 2 Xenva Ltd, George Street, Ryde, Isle of Wight PO33 2JF, UK

## Background

On 21 January 2008 the UK Government's Environmental Audit Committee (EAC) published its report on the inquiry 'Are biofuels sustainable?' [1]. Their short answer, which has since been echoed by a wave of media coverage and environmental group campaigning, was a resounding 'No'. The report concludes that the stimulation of biofuels production by the UK Government and by the EU is reckless. It urges the UK Government to withdraw support for biofuels, and to persuade the EU to do likewise by putting a moratorium on the current 5.75% target for biofuels until more sustainable production processes are developed.

This review argues against this conclusion. Globally, the development of an efficient biofuels industry is an environmental and economic imperative and the UK should leverage its capabilities in life sciences, energy and process industries to help meet this challenge. The EU is right to promote 'sustainable' biofuels through the Renewable Energy Directive, provided that sustainability criteria are implemented effectively and applied consistently.

The remit of the EAC, established in 1997 by the newly elected Labour Government, is to advise the UK Government on the likely impact of current policy on environmental protection and sustainable development. Sixteen members of parliament drawn from across party lines form the current committee, the majority of whom are philosophers, historians and agricultural college graduates, with one scientist (Dr Desmond Turner) thrown in for good measure. Since July 2007, the committee has considered an impressive set of oral and written evidence from research organisations, pressure groups, UK Government departments, industry bodies and corporations.

One week before the EAC's report came out, the Royal Society published its own report 'Sustainable Biofuels: Prospects and Challenges' [2]. The Royal Society arrived at a different conclusion, that biofuels have the potential to be an important part of the future transport energy mix, and can contribute to greenhouse gas (GHG) reduction and energy security subject to two caveats:

1. not all biofuels offer GHG reductions and energy security benefits, and different biofuels must be assessed on their respective merits;

2. this assessment must include agronomic, environmental, economic and social evaluation of the complete cycle including up-front land use changes, and address global and regional impacts, not just local ones.

Like the EAC, The Royal Society's science policy team produce independent advice aimed at influencing UK Government policy. In contrast to the EAC, the Royal Society's working group for this report consisted entirely of scientists from active research organisations, and with relevant expertise. After a 15-month gestation period during which evidence broadly overlapping that submitted to the EAC was considered, the Royal Society's report and its findings were reviewed by a separate panel of leading scientists, and then published.

Who is right?

## Biofuels: a global imperative

The substance of the EAC's argument is as follows: no biofuel is carbon-neutral today. Although GHG emissions from each litre of fuel burned in an internal combustion engine are absorbed by plants grown to produce the next litre, net CO_2 _emissions are released by fossil fuels used in the agricultural, manufacturing and distribution processes that produce the fuel. In the most extreme cases, GHG emissions from fossil fuel inputs can exceed those avoided by the end user. In addition, any forest or grassland that is lost to make way for cultivation of feedstock oil, starch or sugar crops causes an enormous one-off release of carbon dioxide, and the ongoing production of artificially fertilised crops releases nitrous oxide, a GHG almost 300 times more potent than carbon dioxide. Add in the social cost of higher food prices, and the environmental impact of habitat loss, soil erosion and water depletion as intensive biofuel agriculture expands globally, and the arguments against continued political support for biofuels in the UK and EU appear to be overwhelming. Surely we should wait until better alternatives are available, perhaps back the most promising technologies with government funding, and in the mean time look at efficiency measures to curb our transport emissions.

This argument appears persuasive, and is partially based on facts. However, it misses four important points and therefore draws flawed conclusions.

First, we do not have the luxury of time implied by the EAC's 'wait and see' recommendation. Road transport fuels are produced from oil reserves that are being depleted at a rate in excess of new discovery [3]. Global transport demand continues to grow, driven by India and China, and oil scarcity is leading to increasing use of alternative sources of transport fuel such as tar sands, heavy oil, shale and coal-to-liquid processes. As well as being generally more expensive than fuel from conventional oil reserves, these sources are more environmentally damaging both in terms of the local impact of extraction and refining, and in terms of GHG emissions [4]. For example, the extraction of oil from tar sands requires large quantities of steam and the fuel thus produced causes at least 50% more GHG emissions compared with the extraction and use of conventional crude oil. Coal-to-liquid process technology is even less efficient, with almost a third of the coal's chemical energy lost as waste heat in the conversion process. Even the extraction and use of our remaining conventional oil reserves will, in the future, produce higher GHG emissions than today, owing to the smaller size and geographic inaccessibility of the remaining productive fields. This double-whammy of increased demand for more damaging fuels creates an imperative for action. A moratorium on EU biofuel targets as recommended by the EAC implicitly endorses these more polluting alternatives.

Well, is the answer to make us all drive smaller cars that use less oil? The second point missed by the EAC concerns the underlying economics of transport emissions growth. Efficiency is clearly desirable from a consumer's perspective as it lowers the cost of motoring, but will not abate the aggregate growth in transport fossil fuel use. Tata's Nano, unveiled in January as the world's cheapest car, claims an impressive average fuel consumption of 50 mpg [5] yet nobody suspects for a moment that this will reduce India's GHG emissions. Rather, at a price of just over £1000 and with low running costs the Nano is expected to create a transport revolution that will see car use in India soar. Lower price stimulates demand growth. Similarly, any improvement in average fuel economy in the UK or Europe as a whole is unlikely to slow the global rate of oil production and consumption, which it would need to do to have any impact on global emissions. Whether motivated by regulation, environmental concern or thrift, ongoing efforts to improve fuel economy in Europe will be offset by a corresponding increase in consumption elsewhere, until this new demand (stimulated by temporarily lower oil prices) takes up the slack. The only practical way to ensure fossil fuels are left in the ground is to create an abundance of cheaper alternatives. Larry Page, co-founder of Google and champion of Google's renewable energy initiative, recognises this need: "We want to produce one gigawatt of renewable energy capacity that is cheaper than coal. We are optimistic this can be done in years, not decades". Likewise, the goal of a transport biofuels policy must be to create a cheaper and more sustainable alternative to oil as soon as possible.

## Rising to the challenge: industry, policy and technology

The EAC takes the view that the necessary 'second-generation' biofuel technology will be conjured up in a timely fashion, and that until this is available we need not and should not support a biofuel industry based on 'first-generation' technology. Again the EAC is wide of the mark here, as recent experience suggests that the required level of research, development and innovation activity is unlikely happen in the absence of a developing industrial base.

When Cambridge and Imperial College bid against three rival US universities last year for $500 million of funding by BP to establish an Energy Biosciences Institute, the winner was Berkeley. Generous state funding played a part in this decision, but the growing stable of biotechnology and process technology start-ups across North America developing second-generation biofuel technology, financed by mostly non-government capital, bear witness to the commercial attractiveness of this sector.

Investment on this scale depends on the existence of first-generation biofuels, which in the US means ethanol. With an annual output approaching 25 billion litres, the US has overtaken Brazil as the world's largest bioethanol producer [6], and output is expected to double by 2020. The US ethanol industry receives generous state support, and is based on corn feedstock using first-generation technology that is arguably not much better for the environment than burning the oil it displaces. Yet this industry has created an investment climate that has accelerated second-generation technology development. First-generation ethanol plants provide a valuable market for incremental technology in the form of better feedstock varieties, enzymes, microbes and other process enhancements. They stimulate the development of infrastructure: storage facilities, distribution networks and flex-fuel vehicles necessary for future bioethanol consumption growth. They also create the experience and know-how that venture capital investors look for in start-up management teams.

The marginal environmental benefits of corn ethanol are mirrored by marginal economics, and in the past year rising feedstock and energy costs have left many US plants operating at or below breakeven. It is precisely for this reason that improvements in process technology are so valuable to the industry, and why the global centre of second-generation bioethanol research and expertise has developed in North America, not in Brazil where first-generation economics are perfectly adequate. Government funding aside, BP's decision to locate its Energy Biosciences Institute in California recognises this expertise, and that for the US bioethanol industry, economic necessity is the mother of invention.

Like the US, the UK is a world leader in life sciences and in process engineering. Unlike the US, we have a relatively poor track record when it comes to commercialising these capabilities, which require clear policy incentives, regulatory frameworks and a solid industrial base in which to develop. The number of bioethanol technology start-ups based here today can be counted on the fingers of one hand. This is likely to remain the case until the UK has an established and credible bioethanol production industry with commercial needs and money to spend.

The final point missed by the EAC when it asserts that "The [UK] government should concentrate on the development of more efficient biofuel technologies that might have a sustainable role in future" concerns the ability of government to pick winners when it comes to emerging and potentially disruptive technology [7]. Current biofuel research is active in three overlapping areas: feedstock development, process improvement and new process design. In each area, a large number of public, corporate and start-up organisations are racing to bring new technologies to market. In some cases these are incremental and near term, but a number of technologies in development have potential to transform the entire global industry, any one of these may emerge as the prevailing biofuel process route in the future. Biodiesel made from fast-growing algae, enzyme hydrolysis of forest waste and grasses, thermal depolymerisation of organic waste to form 'biocrude', and even direct biological synthesis of more complex biofuels each have such potential. Each may also be rendered impotent by an insurmountable technical or economic hurdle. Most innovation fails, and ultimately only those technologies that offer better returns on available resources are adopted.

In an efficient market for research funding, many early stage technologies that show promise receive investment, and most die when their potential is shown to be flawed or if a more promising competing alternative shows up. Government-funded research, in contrast, tends to be concentrated in projects where there is also political capital to be made, once initial investment is sunk it becomes increasingly difficult for support to be withdrawn without the damaging suggestion that funds have been wasted or promises broken. It often ends up backing the wrong technology.

When the US Department of Energy (DoE) announced a $1 billion public-private clean-coal technology demonstration plant known as 'FutureGen' in 2003, this was positioned as a 'living prototype' centrepiece to the Bush administration's carbon sequestration leadership programme, singled out by the President himself as one of the most promising approaches for reducing GHG emissions. Then, in January 2008, energy secretary Sam Bodman announced that further government funding would be withdrawn, to howls of protest from stakeholders in the project. Instead, the DoE will support a number of bolt-on clean-coal projects that will add third-party carbon sequestration technology to existing plants. This technology, developed elsewhere since the FutureGen project began, is considered a more efficient use of public funds. In other words "We backed the wrong horse".

Government research and development accounts for less than 15% of global investment in alternative energy [8], and in Europe this figure is lower still. The likelihood, in Europe at least, is that the majority of biofuel technology improvements that successfully deliver environmental policy objectives will be developed by the private sector in response to clear policy signals, and closely aligned with the needs of a commercially viable biofuel industry. The key role for policy is to create sufficient up-side potential both for producers and technology developers to warrant the high levels of investment necessary for innovation and advancement towards the most efficient, and sustainable, use of resources. This was a conclusion of the Royal Society's report: "There is an urgent need for policies that promote the commercialization of biofuels and stimulate the development of new technologies that are efficient and environmentally beneficial. In particular, industry needs clear and coherent policy signals that provide a long-term favourable framework for development. An obvious step would be to extend the Renewable Transport Fuels Obligation or the fuel duty allowance to 2025".

This is not to dismiss the significant social and environmental problems associated with some biofuels today, but it is in stark contrast to the recommendation of the EAC, that "biofuels from conventional crops should no longer receive support from the UK and EU governments", which, if adopted, would substantially delay the point at which the commercially led investment needed to address these problems would begin to flow in Europe.

## A European opportunity

Of all of the problems flagged by the EAC, the impact of biofuel growth on land use is by far the most significant for GHG emissions. In 2000, agriculture and land use change together represented 32% of global GHG emissions, compared with just 14% for transport [9]. Land use GHG emissions are mostly down to deforestation for agriculture, and half are generated by land use changes in just two countries: Brazil and Indonesia [10]. In both cases agricultural expansion is being driven by the booming demand for biofuel feedstocks: sugar cane in Brazil and palm oil in Indonesia. Brazilian bioethanol is often regarded as the gold standard in renewable fuels, because its production and distribution saves around 10 times the fossil fuel inputs required, compared with less than twice the saving for corn ethanol in the US. Yet if this biofuel production causes deforestation, whether directly or indirectly through the displacement of other agricultural land use, the one-off GHG release during deforestation could take over 20 years to offset by growing sugar cane feedstock for bioethanol on the same land. Payback for land deforested for palm oil takes even longer, by some estimates this could be 400 years or more [11,12].

Biofuel produced from deforested land is clearly not sustainable, and therefore as both the EAC and Royal Society recognise, each biofuel must be assessed on its own merits including the impact of land use change. The question is, until differentiation based on this assessment is possible, should we promote the development and use of all biofuels, a select few or, as the EAC recommends, none at all?

Under current UK policy the Renewable Transport Fuel Obligation (RTFO) will be supported by a proposed carbon reporting and sustainability certification scheme, and incentives will be based on the carbon performance of the biofuel used, taking the feedstock source, processing and distribution into account [13]. The EAC states in no uncertain terms that this is not good enough, and that UK and EU targets for biofuel use should be suspended until adequate sustainability standards are in place.

This hard-line conclusion draws heavily on views expressed in environmental pressure group inputs to the inquiry. These include witness evidence from the WWF, Friends of the Earth and the Royal Society for the Protection of Birds (which counts several of the inquiry's committee amongst its members) and whose justifiable concern at the global habitat loss caused by deforestation and other land use changes found an outlet in this inquiry.

The effect of increased biofuel production from feedstocks grown within Europe cannot be likened to the devastating impact of palm-oil plantations that now roll across former Indonesian rainforests. Since the EU's Biofuels Directive was adopted in May 2003, EU biofuel production has more than doubled every two years [14]. Yet Europe's forests are not shrinking, but growing at about 0.4% annually as they have done since 1990 and represent almost twice the total area of Europe's arable farmland [15]. Biofuel feedstock production in Europe taps into two substantial resources: Eight million hectares of set-aside land, and productivity improvements through better plant varieties and agronomic practices. Productivity gains, particularly in the new member states in Eastern Europe, represent the larger of these resources. Several countries here currently achieve less than half the wheat yield compared with those from similar soils and climatic conditions in Western Europe, whereas East Germany's 'yield gap' has been closed since reunification and wheat yields, now similar to those achieved in West Germany, are amongst the highest in Europe [16].

Other arguments against the sustainability of biofuels in Europe raised by the EAC do not stand up to scrutiny. The economic case against biofuels cited by the EAC gives an uneconomic CO_2 _abatement cost for wheat bioethanol of £152/tonne and £137/tonne for biodiesel, supported by data sourced from the 2007 DTI paper 'UK Biomass Strategy' [17]. Yet on closer inspection, the DTI paper actually qualifies this data as 'illustrative'. Moreover, the DTI analysis dates back to 2005 and uses a projected oil cost of $40/barrel. True GHG abatement costs of biofuel incentives remain difficult to evaluate with any accuracy, as different production methods used for the same feedstock have a large impact, but agricultural GHG emissions can be reduced through known practices such as low-till cultivation to minimise soil CO_2 _losses [18], selective application of fertiliser in response to plant demand to minimise N_2_O emissions [19], and by new approaches such as application of 'Biochar' nutrients that sequester carbon whilst improving soil fertility [20]. Given that most agriculture is for food production, the right way to tackle this issue is through policy that controls all agricultural supply, rather than just biofuel feedstocks. The EU's single farm payment scheme already stipulates good environmental management practices that include control of chemical and fertiliser use, set-aside areas, field boundary sizes and highly specific measures such as patches within fields set aside for ground nesting birds. This scheme could also provide a mechanism for managing down the overall GHG contribution of European agriculture.

Perhaps the most emotive argument against sustainable biofuel production in Europe is 'food for fuel', that the diversion of agricultural output into biofuel production will drive up prices and deprive the world's poor of food. The EAC quote Jean Zeagler of the UN: " [It is] a crime against humanity to divert arable land to the production of crops which are then burned for fuel". This is an argument that resonates with the public awareness of rising food prices in supermarkets, particularly for foods with wheat- and corn-based ingredients, but again, here, all is not what it seems. Global wheat prices in real terms today remain well below their historic levels until the mid 1980s, when agricultural policies subsidising production in the EU and US conspired to create a global grain mountain [21]. This surplus depressed free market prices and led to massive flows of low-priced agricultural exports to developing countries, destroying the livelihoods of local farmers and stifling agricultural development. This is a bone of contention that continued to dog EU agricultural policy in 2006 according to Claire Godfrey, trade policy adviser for Oxfam: "Not only does the Common Agricultural Policy hit European shoppers in their pockets but strikes a blow against the heart of development in places like Africa. The UK government must lobby hard within the EU to agree an overhaul of the Common Agricultural Policy by 2008 to put an end to the vicious cycle of overproduction and dumping." [22].

In practice, the 'food for fuel' argument can be applied to any use of agricultural resources whose output does not directly feed people. Of these, the most significant are the production of animal feed and the production of biofuel feedstocks. Global growth in demand for animal feed is accelerating due to the combined impact of population increase and increased per capita income, which drives up consumption of meat. In Europe over 140 million tonnes, more than half of all grain produced, is used for animal feed each year [23] and a further 45 million tonnes of oil meals are imported to make up the necessary supply of animal feed protein [24].

What the EAC failed to acknowledge in its consideration of the impact of biofuels on food security is that a co-product of both bioethanol and biodiesel production is high-protein animal feed. The UK Government's Advisory Committee on Animal Feedingstuffs considered in 2007 whether biofuels would drive up the price of animal feed in the EU, and concluded that impact would be neutral, with any increase in grain prices offset by an increased supply of feed co-produced from biofuel processes [25].

## The way forward

A more defensible conclusion from consideration of the EAC's evidence under review might be to propose a moratorium on EU imports of biofuels and certain feedstocks, whilst maintaining proportionate and long-term binding targets for biofuels produced in Europe. Europe's nascent biofuel industry might then provide a commercial environment capable of supporting development and implementation of the necessary technology and regulation for a truly sustainable European biofuel industry.

The obstacle to such an approach is that it is open to challenge by the World Trade Organization (WTO) and by potential biofuel-exporting nations as 'green protectionism'. Any sustainability criteria must therefore apply equally to European producers and to overseas producers wishing to export feedstocks or biofuels to the EU market. This, as it happens, is precisely what the EU intends to do. On 23 January 2008 shortly after the EAC's report, the European Commission published its proposal for a directive on the promotion of the use of energy from renewable sources [26] that sides firmly with the conclusions of the Royal Society. This proposal, if adopted, will supersede the 2003 Biofuels Directive and introduce a 10% binding target for biofuels by energy content for all member states by 2020. Only those biofuels that meet a range of sustainability criteria and achieve a 35% minimum greenhouse gas saving (including the impact of land use change) will count towards this target. To encourage more efficient biofuel technology development, cellulosic and waste-based biofuels will count towards a country's overall target twice. The clarity, binding nature and long-term framework for sustainable growth set out in this proposal have received the backing of many of the leading players in the EU's biofuel industry. The EU proposal has the merits of providing a policy framework that will stimulate industrial innovation, whilst differentially rewarding those technologies and producers that most effectively address the valid sustainability concerns raised by many observers of the global biofuels industry today.

The UK Government relies on good scientific advice to help formulate policy that best meets the challenges anticipated in an uncertain future. As we enter final few decades of oil, UK, EU and international policy will influence the rate at which remaining reserves are consumed, what alternatives will be used in their place and how costly the transition will be: socially, economically and environmentally. 'Wait and see' is not good enough. What the EAC is proposing is neither precautionary nor progressive, but is based on emotive and flawed arguments that, whilst delivered with conviction, are supported neither by the evidence considered nor by the broader scientific and economic data available.

The UK Government should set the EAC's report aside and back the EU proposal.

## References

[B1] EAC (2008). Are Biofuels Sustainable?.

[B2] The Royal Society (2008). Sustainable Biofuels: Prospects and Challenges.

[B3] Global BP (2007). Statistical review of world energy. http://www.bp.com.

[B4] Farrell AE, Brandt AR (2006). Risks of the oil transition. Environ Res Lett.

[B5] O'Connor A (2008). Tata Nano – world's cheapest new car is unveiled in India. Times Online.

[B6] F.O. Licht (2007). World Ethanol Biofuels Rep 23 October.

[B7] Gill D, Minshall T, Pickering C, Rigby M (2007). Funding Technology: Britain Forty Years On.

[B8] Goldman Sachs (2007). Alternative Energy: A Global Survey. http://www.gs.com/gmi/.

[B9] World Resources Institute (2008). Climate Analysis Indicators Tool version 5.0. http://cait.wri.org/.

[B10] Stern N (2007). The Economics of Climate Change.

[B11] Fargione J, Hill J, Tilman D, Polasky S, Hawthorne P (2008). Land clearing and the biofuel carbon debt. Science.

[B12] Giles J (2008). Biofuels emissions may be 'worse than petrol'. New Scientist.

[B13] Low Carbon Vehicle Partnership (2006). Development of Sustainability Reporting as part of the RTFO.

[B14] European Commission (2006). EU Policy for Energy from Biomass.

[B15] FAO (2007). State of the World's Forests.

[B16] USDA Foreign Agricultural Service (2003). Eastern Germany Crop Yield Increases Herald Changes in Central Europe.

[B17] DTI (2007). UK Biomass Strategy Working Paper 1 – Economic Analysis of Biomass Energy.

[B18] Manley J, van Kooten G, Moeltner K, Johnson D (2005). Creating carbon offsets in agriculture through no-till cultivation: a meta-analysis of costs and carbon benefits. Climatic Change.

[B19] WBCSD (2002). Norsk Hydro: Farmer Decision Support Tools.

[B20] Lehmann J (2007). Bio-energy in the black. Front Ecol Environ.

[B21] UNCTAD (2006). Handbook of statistics. http://www.unctad.org/statistics/handbook.

[B22] Frith M (2006). EU subsidies deny Africa's farmers of their livelihood. The Independent.

[B23] Hemeline M Bio energy – present situation in the EU27 and the effect on the grain market for the coming years. Proceedings of Global Grain: 21–23 November 2007; Geneva.

[B24] FAO (2007). Food Outlook.

[B25] ACAF (2007). Information Paper ACAF/07/24.

[B26] European Commission (2008). Energy and climate change:promotion of the use of energy from renewable sources RES-E. Directive on the Promotion of the Use of Energy from Renewable Sources COD/2008/0016.

